# What Is Recovery?

**DOI:** 10.35946/arcr.v40.3.01

**Published:** 2020-09-24

**Authors:** Katie Witkiewitz, Kevin S. Montes, Frank J. Schwebel, Jalie A. Tucker

**Affiliations:** 1 Department of Psychology, University of New Mexico, Albuquerque, New Mexico; 2 Center on Alcohol, Substance Use, and Addictions, University of New Mexico, Albuquerque, New Mexico; 3 Department of Psychology, California State University Dominguez Hills, Carson, California; 4 Department of Health Education and Behavior and Center for Behavioral Economic Health Research, University of Florida, Gainesville, Florida

**Keywords:** recovery, alcohol use disorder, alcohol dependence, remission, life-health-functioning, alcohol consumption, alcohol

## Abstract

Alcohol use disorder (AUD) is among the most prevalent psychiatric disorders and is associated with enormous public health costs. Although AUD and other addictive behaviors have been described as chronic relapsing conditions, most individuals who develop AUD will eventually recover. This narrative review provides an overview of definitions of recovery, with a focus on recovery from AUD. The definitions reviewed include those developed by key stakeholder groups, as well as definitions derived from recent quantitative and qualitative studies of individuals who meet criteria for AUD and attempt to resolve their problems with or without treatment or who self-identify as pursuing or achieving recovery. The literature reviewed supports a definition of recovery as an ongoing dynamic process of behavior change characterized by relatively stable improvements in biopsychosocial functioning and purpose in life. The review concludes that definitions of recovery that rely solely on abstinence from alcohol and the absence of AUD symptoms fail to capture the multidimensional and heterogeneous pathways to recovery that are evident among individuals in general population and clinical samples.

## INTRODUCTION

Alcohol use is associated with tremendous social and economic costs and contributes to 5% of the global disease burden.^1^ Most of the costs are due to excessive drinking and alcohol use disorder (AUD), with AUD defined by the fifth edition of the *Diagnostic and Statistical Manual for Mental Disorders* (DSM-5) as clinically significant impairment or distress resulting from endorsing at least two of 11 symptoms in the past 12 months.^2^ Based on epidemiological survey data in the United States, as many as 14% of individuals meet criteria for current AUD, and nearly one-third (29%) meet lifetime criteria for AUD.^3^ Importantly, data from national epidemiological surveys, prospective observational studies, and randomized clinical trials of patients with AUD and individuals who engage in problem use of alcohol indicate that most affected persons will ultimately recover^4^—with “recovery” defined in various ways by different stakeholders including researchers, clinicians, mutual help groups, health care organizations and policymakers, and persons with AUD.

Defining recovery consistently across studies and by various stakeholder groups is critical for advancing the science of AUD. First, through an agreed-upon definition of recovery, a better understanding can be gained of the clinical course of AUD and how AUD symptoms change over time. Second, an agreed-upon definition will facilitate the evaluation and dissemination of treatments for AUD, thereby increasing understanding of which treatments are associated with shorter- versus longer-term recovery from AUD and guiding development of new treatments to offer recovery support. Third, a definition of recovery will help individuals with AUD and their family and friends, health care providers and organizations, and policymakers gain a better understanding of the process of change in AUD and will help clarify expectations about change goals during the process of change. Fourth, operationalizing recovery may help to reduce the stigma associated with AUD by highlighting its possibility and prevalence and by providing both hope and a positive characterization of the AUD recovery process.^5,6^

The goals of this narrative review are to examine historical and current definitions of recovery, which are variable across studies and stakeholders; to review recent quantitative, qualitative, and mixed-methods studies that have examined the recovery construct among individuals with AUD; and to provide a new conceptual definition of recovery that is based on recent empirical findings. The discussion begins with an overview of the major diagnostic systems developed by the American Psychiatric Association in DSM-5 and the World Health Organization *International Classification of Diseases* (ICD-10) and the definitions of AUD and remission based on those systems. Historical definitions of recovery are then reviewed as defined by the Temperance Movement, early medical literature, the “Big Book” of Alcoholics Anonymous,^7^ and the early behavior therapy movement. Current definitions of recovery as proposed by key stakeholder groups are considered next, followed by consideration of findings from quantitative and qualitative research that informs definitions of recovery among individuals who are attempting to resolve alcohol-related problems with or without formal treatment and who do and do not identify as being in or achieving recovery. A final section concludes with a summary of common themes across definitions and proposes an expanded definition of recovery that emphasizes improvements in well-being and functioning.

## CLINICAL DIAGNOSIS OF AUD

DSM-5 defines AUD based on meeting criteria for two of 11 symptoms in the past 12 months.^2^ The 11 symptoms can be roughly organized into four symptom clusters:

- Physiological correlates of alcohol use— (1) tolerance, (2) craving, and (3) symptoms of withdrawal;
- Loss of control over alcohol use—(4) drinking longer or larger amounts than intended, and (5) unsuccessful efforts to cut down or control drinking;
- Alcohol taking over other meaningful activities—(6) time spent in activities related to alcohol, and (7) other activities given up because of alcohol; and
- Problems resulting from alcohol use— (8) failure to fulfill role obligations, (9) social or interpersonal problems, (10) physical or psychological problems, and (11) use in situations that are physically hazardous.

DSM-5 also provides a definition of remission from AUD based on the length of time that symptoms are no longer present. Early remission is defined as greater than 3 months and less than 12 months of endorsing no symptoms of AUD, with the exception of craving. Sustained remission is defined as 12 months or more of endorsing no symptoms of AUD, with the exception of craving. Craving is excluded from definitions of remission given that craving could persist long after remission of other AUD symptoms is achieved.^8^

ICD-10 defined alcohol dependence based on meeting three or more of six symptoms in the past 12 months, including (1) tolerance, (2) craving, (3) physiological withdrawal, (4) loss of control, (5) alcohol taking over other activities, and (6) problems resulting from alcohol use.^9^ ICD-11 defines alcohol dependence as endorsement of two of three core features in the past 12 months, including (1) impaired control over alcohol, often including craving; (2) alcohol becomes increasingly prioritized in life, often despite problems; and (3) physiological features caused by pharmacological tolerance and withdrawal.^10^ ICD-11 also includes codes for early full remission, defined as abstinence from alcohol lasting 1 to 12 months; sustained partial remission, defined as “significant reduction in alcohol consumption for more than 12 months” and not meeting criteria for ICD-11 alcohol dependence; and sustained full remission, defined as abstinence from alcohol lasting 12 months or longer.^11^ Thus, according to ICD-11, full remission (early or sustained) requires abstinence from alcohol, and partial remission is defined by reductions in drinking and the absence of symptoms of disorder. In contrast, as noted above, the DSM-5 definition of remission is based solely on not meeting symptoms of the disorder and does not consider alcohol consumption.

## DEFINITIONS OF RECOVERY

### Historical Perspectives and Definitions of Recovery

As early as the late 1700s, American physician Benjamin Rush wrote about the effects of alcohol on the human body and mind, as well as potential remedies for “curing the ardent use of spirits on the body and mind.”^12^ Rush noted that abstinence from liquor was critical, while allowing consumption of larger quantities of beer or wine as acceptable substitutes for liquor. He concluded: “By the temporary use of these substitutes for spirits, I have never known the transition to sober habits, to be attended with any bad effects but often with permanent health of body, and peace of mind” (p. 32).

This very early harm reduction perspective contrasts with the subsequent focus of the Temperance Movement on ridding society of alcohol. The movement was active through the remainder of the 1800s and into the early 1900s and had many distinct groups and societies. Initially, the Temperance Movement focused on promoting abstinence from liquor, then transitioned to a singular goal of abstinence from alcohol, and later advocated for the legal prohibition of alcohol.^14^ Inebriate asylums, which required abstinence from alcohol, emerged as a residential treatment option in the 1840s.^13^

The Temperance Movement was followed by the founding of Alcoholics Anonymous (AA) in the 1930s,^14^ and AA has since had tremendous influence on modern conceptualizations of recovery. AA proposed a mutual help program defined by a 12-step recovery process for achieving and maintaining lifelong abstinence from alcohol. The “Big Book” of AA, first published in 1939, also made very clear that abstinence from alcohol was not sufficient to define recovery.^7^ The Big Book describes the process of recovery through many of the chapters as a journey that includes major transformative changes that lead to improvements in health, functioning, and well-being.^7^ Most of the 12 steps focus on addressing and resolving past and present problems associated with “alcoholism,” a term first used by Swedish physician Magnus Huss in the mid-1800s.

In the mid-20th century, biostatistician and physiologist E. M. Jellinek led several initiatives aimed at increasing the study and dissemination of science related to “alcoholism,” including early work studying members of AA and patients in treatment. Jellinek also proposed the disease concept of alcoholism, which he characterized as a progressive and chronic disease with several variants or “species.”).^15^^(pp154–158)^ Glatt expanded on Jellinek’s model by developing a plan for rehabilitation and remission through a group treatment program largely based on AA principles and practices.^16^ Early work by Edwards further helped define the disease concept,^17^ and pioneering work by Vaillant shed light on the possibility that individuals with AUD could recover in the absence of treatment.^18^ Thus, early work was heavily influenced by AA, and abstinence was generally considered critical to recovery until the late 1900s.

In the 1970s, psychiatrist Mansell Pattison and psychologists Mark and Linda Sobell introduced modern behavioral conceptualizations of alcohol dependence that have replaced the disease concept of alcohol dependence in research and evidence-based treatments.^19^ They defined alcohol dependence as a serious health problem “defined by drinking patterns and the adverse physical, psychological and/or social consequences of such drinking”; considered patterns of alcohol use as “lying on a continuum ranging from non-pathological to severely pathological” and noted that problem development “follows variable patterns over time and does not necessarily proceed inexorably to severe fatal stages;” and concluded that “[r]ecovery from alcohol dependence bears no necessary relation to abstinence, although such a concurrence is frequently the case” (pp. 4–5). This seminal reconceptualization of alcohol dependence and recovery remains relevant and influential in current research on AUD today. It was foundational in behavior therapy research and practice beginning in the 1970s to the present, a movement that produced evidence-based treatments in use today, including relapse prevention, motivational interviewing, reinforcement-based treatments, and cognitive behavioral therapy for AUD.

Also in the 1970s, the Sobells’ clinical research demonstrating controlled drinking outcomes (defined as drinking fewer than 4.3 standard drinks on most days with allowance of up to 6.5 drinks for an isolated 1- or 2-day sequence) among a subset of treated patients with alcohol dependence sparked virulent controversy and challenged the then dominant view that recovery required lifelong abstinence.^20^ Subsequent research has replicated and extended their findings.^21^ Although specific quantity/frequency criteria used to define low- versus high-risk drinking practices are somewhat variable across studies and countries, low-risk drinking is now well established as a favorable outcome among persons previously diagnosed with AUD. For example, in the United States, low-risk drinking has been defined as consumption of fewer than 14 drinks per week with fewer than four drinks on any given day for men and fewer than seven drinks per week with fewer than three drinks on any given day for women. In contrast, consumption criteria considered indicative of higher-risk drinking practices are any occasions of more than 14 drinks weekly or more than five drinks daily for men and more than seven drinks weekly or more than four drinks daily for women.^22^ As discussed in the rest of this paper and elsewhere,^4,23^ these criteria have been widely adopted in recovery research, but have been found wanting as an outcome metric on several grounds and have contributed to a lesser focus on measures of well-being and functioning, which are central to most current definitions of recovery.

### Current Definitions of Recovery

Recent illustrative definitions of recovery (summarized in Table 1) have focused on the importance of functioning and general well-being in defining recovery. For example, the Substance Abuse and Mental Health Services Administration (SAMHSA) advanced a working definition of recovery as “a process of change through which individuals improve their health and wellness, live a self-directed life, and strive to reach their full potential.”^5^ SAMHSA noted the importance of abstinence as one example of achieving improvements in health. Similarly, the Betty Ford Institute Consensus Panel in 2007 defined recovery as “a voluntarily maintained lifestyle characterized by sobriety, personal health, and citizenship.”^24^^(p222)^ Similar to the Big Book of AA, these definitions acknowledge that abstinence is not a sufficient condition for recovery and that an individual who merely abstains from alcohol, with little or no improvement in functioning or well-being, would not be considered to be in recovery. In 2017, a Recovery Science Research Collaborative meeting was convened by recovery researchers with a specific focus on examining the concept of recovery based on a literature review and ideas generated by group members.^25^ Their final definition was: “Recovery is an individualized, intentional, dynamic, and relational process involving sustained efforts to improve wellness.” ^25^^(p5)^ This definition acknowledges the presence and importance of individual differences in the recovery process; it focuses on the recovery process as being both intentional and dynamic and as requiring sustained efforts to improve wellness. Improving wellness includes not only the physical benefits associated with reducing alcohol use,^26^ but also benefits related to psychosocial and functional dimensions of wellness (e.g., social, emotional, financial).^27^

On balance, similar to AA’s view that recovery is optimally broad in scope, these recent consensus definitions of recovery focus heavily on enhanced well-being and functional improvements in areas adversely affected by drinking. They do not emphasize or are silent about changes in drinking or achieving abstinence. These characterizations, as well as recent empirical research on AUD recovery (described next), are similar to definitions of recovery for other psychiatric disorders (e.g., depression, schizophrenia) that emphasize recovery of functioning and do not require absence of any symptoms. These definitions differ from definitions of recovery from other health conditions such as cancer, that do not require improvement in well-being and quality of life.

## EMPIRICAL RESEARCH EXAMINING RECOVERY AMONG INDIVIDUALS WITH AUD

### Recent Quantitative Research on Recovery

As summarized by Tucker et al., research using both clinical and non-treatment-seeking samples has shown that the majority of individuals who develop AUD reduce or resolve their problem over time.^4^ The pathways to improvement are heterogeneous, may occur with or without participation in treatment or mutual help groups, and involve improved functioning and well-being with or without reductions in drinking. Several lines of quantitative research, ranging from treatment outcome to naturalistic observational studies, have converged to support this expanded characterization of improvement in alcohol-related problems. Collectively, this body of work questions conventional views that alcohol and other drug use disorders are “chronically relapsing” conditions, for which treatment or mutual help group involvement is essential for recovery.^28,29^

For example, using data-driven approaches to studying longer-term outcomes among individuals with AUD who enrolled in clinical trials targeting AUD, Witkiewitz and colleagues followed treatment recipients for 3 years and identified four profiles of individuals based on intensity and frequency of alcohol consumption, as well as other indicators of health and well-being: (1) low-functioning frequent heavy drinkers, (2) low-functioning infrequent heavy drinkers, (3) high-functioning occasional heavy drinkers, and (4) high-functioning infrequent non–heavy drinkers.^30^ Relative to high-functioning infrequent non–heavy drinkers, individuals who were high-functioning occasional heavy drinkers had lower baseline alcohol dependence severity, lower abstinence self-efficacy, and lower AA involvement, but they did not differ on other measures of functioning. High-functioning occasional heavy drinkers had significantly higher purpose in life compared to poor-functioning profiles and greater satisfaction with life compared to abstainers. Beyond portraying a broader representation of AUD outcomes to include both consumption and functioning, this work also helped clarify factors that may contribute to both consumption and functional outcomes. At baseline, greater social support for drinking predicted heavier drinking. Better mental health— including less severe psychiatric symptoms, depression, and anger—and greater purpose in life at 1 year following treatment were significantly associated with higher functioning at 3 years following treatment. Social support at 3 years following treatment was also greatest among the higher-functioning profiles. These findings were recently replicated in an independent sample.^31^

Using a similar data-driven approach, Witbrodt and colleagues identified five latent classes based on recovery elements reported in in-depth interviews and surveys completed by 9,341 individuals who self-identified as being in recovery. The five classes were characterized as (1) 12-step traditionalist, (2) 12-step enthusiast, (3) secular, (4) self-reliant, and (5) atypical.^6,32^ Individuals in the 12-step traditionalist and enthusiast classes were most likely to have been or to be currently engaged in AA or other 12-step programs and were mostly abstinent. Those in the secular, self-reliant, and atypical recovery classes were less likely to be abstinent or engaged in 12-step programs. Across all five classes, four items were commonly endorsed from among the top 10 ranking items as important to recovery: (1) being honest with oneself, (2) handling negative feelings without using drugs or alcohol, (3) being able to enjoy life, and (4) engaging in a process of growth and development.

In prospective research that followed a community sample of individuals who drank alcohol over 20 years, Moos and colleagues found that cognitions, attitudes, and beliefs, as well as contextual, social, and environmental factors, were critically important in predicting long-term reductions in drinking.^33,34^ In terms of the role of drinking, any drinking was not predictive of long-term negative outcomes, but persistent average heavy drinking and heavy episodic drinking were each associated with greater problems related to alcohol use.^35^

Natural recovery studies also have highlighted the role of contextual variables in different pathways to AUD resolution. Tucker and colleagues conducted a series of studies guided by behavioral economics among individuals with AUD who resolved a drinking problem in the absence of treatment.^36,37^ In addition to showing that many participants maintained stable abstinence or low-risk drinking without problems over 1- to 2-year follow-ups, this research distinguished those who maintained low-risk drinking from other outcome groups by how they handled their monetary spending before and after they initially stopped problem drinking (i.e., pre-resolution). Pre-resolution, participants who achieved stable low-risk drinking outcomes had more balanced allocations between spending on alcohol versus saving money for the future compared to those who remained abstinent or relapsed and who spent proportionately more on alcohol than savings. After initial resolution, the spending patterns of stable low-risk drinkers changed in ways that led to receipt of heretofore delayed large rewards (housing in particular) that yielded ongoing lifestyle benefits. By comparison, after resolution, participants who remained abstinent or relapsed spent less overall and tended to spend on smaller rewards (e.g., consumable goods, entertainment, gifts) throughout the post-resolution year, appearing to substitute alcohol with small frequent substance-free rewards. Thus, different recovery-relevant outcomes were associated with patterns and contexts of non-drinking behaviors before and after a quit attempt.

Another issue informed by recent quantitative research concerns the typical number of quit attempts before recovery is achieved. Kelly and colleagues surveyed a national sample of adults in the United States who successfully resolved a significant substance use problem and assessed the number of prior recovery attempts and the relationships between recovery attempts and post-recovery measures of psychological well-being and quality of life.^38^ The mean, median, and modal numbers of recovery attempts were 5.4, 2.0, and 1.0, respectively; however, the distribution was positively skewed and included outliers, suggesting that a subgroup of individuals require many more attempts to change than others and may require a higher level of care. Another subset of participants reported not making a prior serious change attempt. These results are similar to another arm of the National Recovery Study, which reported reasons why individuals did not adopt or dropped the label “recovery” (e.g., putting problem behind them, perceiving low problem severity).^39^

Collectively, these studies support adoption of a more flexible definition of recovery (or other inclusive term) that focuses on improvements in areas of functioning adversely affected by drinking and enhanced access to non-drinking rewards. Furthermore, beneficial changes in limited areas of alcohol-related dysfunction and reductions in drinking can occur that contribute to improved health and well-being, even if they fall short of traditional definitions of recovery that emphasize abstinence as a required element. Although recent research is consistent in supporting these conclusions, they are advanced preliminarily, given that each of the aforementioned findings are from single studies and require additional investigation to establish their robustness and generalizability across diverse AUD populations.

### Recent Qualitative and Mixed-Methods Research on Recovery

Mixed-methods research in the United States and the United Kingdom has elucidated elements of recovery from the perspective of persons seeking to resolve AUD, and findings show consistencies with quantitative research on recovery. For example, Kaskutas and colleagues surveyed 9,341 individuals who self-identified as being in recovery to delineate specific aspects of recovery from the perspective of persons engaged in the process.^6^ The survey consisted of 47 elements of recovery developed via initial qualitative work, which participants rated as (1) definitely belonging in their definition of recovery; (2) somewhat belonging in their definition of recovery; (3) not belonging in their definition, but potentially belonging in others’ definitions of recovery; and (4) not belonging in a definition of recovery. Based on exploratory and confirmatory factor analyses, 35 elements were retained, and a four-factor solution emerged: (1) abstinence, (2) essentials of recovery, (3) enriched recovery, and (4) spirituality in recovery. The “essentials of recovery” factor refers to ways of being considered crucial to maintaining changes in alcohol and drug use (e.g., dealing with challenging negative feelings, realistic self-appraisal). This factor is distinct from the “enriched recovery” factor, which refers to an individual’s ability to look inward (e.g., inner peace) and outward (e.g., living a life that contributes to others and society) and to engage in self-care. The six elements endorsed by more than 90% of participants as definitely belonging to their recovery definition were classified in the “essential recovery” and “enriched recovery” factors and were not in the “abstinence” factor.

Neale and colleagues developed a new patient-reported outcome measure of recovery from drug and alcohol dependence, named the Substance Use Recovery Evaluator (SURE), which incorporates input from addiction psychiatrists and staff as well as individuals in recovery (e.g., former and current users of drug and alcohol services).^40^ Based on exploratory and confirmatory factor analyses, 21 items were retained, and a five-factor solution emerged: (1) substance use, (2) material resources, (3) outlook on life, (4) se lf-care, and (5) relationships. Similar to the findings of Kaskutas and colleagues,^6^ only six of the 21 items pertained specifically to substance use–related recovery outcomes.^40^ SURE provides a patient-centered method to assess a broad range of recovery-related outcomes valued and experienced by those who embark on various pathways toward recovery.

In addition to recent efforts to understand the concept of recovery from the perspective of persons attempting it, another body of research has investigated mechanisms of behavior change that may help explain how individuals are able to recover. For example, Best and colleagues developed the Social Identity Model of Recovery,^41^ which, when applied to alcohol recovery, posits that an individual’s social identity shifts during recovery and becomes defined more by the norms and behaviors of individuals who do not use alcohol (e.g., family members, spouse, friends, members of AA) than by those who drink alcohol. Research on AA has similarly shown that higher rates of AA attendance are associated with greater rates of abstinence and with reporting having more non-drinking friends.^42^ AA engagement also has been found to be a catalyst for social network change that facilitates recovery.^43^

These findings highlight how changes in one’s social identity and social network may support AUD recovery. Further investigation of social identity models, the role of social networks, and patient-centered research on the recovery experience is important for broadening the scope of assessment of recovery-relevant outcomes. In addition to contributing knowledge about how people recover, such qualitative research can inform improvements in alcohol services that are responsive to the preferences and needs of consumers of services and thus may help close the long-standing gap between need and alcohol services utilization.

## WHAT IS RECOVERY? CONCEPTUALIZATION AND FUTURE DIRECTIONS FOR RESEARCH AND PRACTICE

Drawing from prior definitions and informed by recent empirical work, the authors conclude that recovery is a process of behavior change characterized by improvements in biopsychosocial functioning and purpose in life. As shown in Table 1, this conceptualization of recovery is similar to definitions of recovery developed by SAMHSA and the Recovery Science Research Collaborative, and it aligns with the empirical findings from Kaskutas, Neale, Kelly, and Witkiewitz, among others. These conceptualizations of recovery, including that of the authors, differ from the Betty Ford Institute Consensus Panel, which requires abstinence. Similarities across definitions of recovery shown in Table 1 indicate that alcohol recovery is a process that is dynamic and focuses on improvement of health and wellness. Definitions differ with respect to the inclusion of language pertaining to abstinence or changes and improvement in biopsychosocial functioning and purpose in life.

Based on the available literature, the authors question the validity of any definitions of recovery that rely solely on abstinence from alcohol or the absence of AUD symptoms and fail to consider changes in other outcomes related to improved functioning and purpose in life. Abstinence will be important for some individuals to start the recovery process and will likely contribute to the abatement of many AUD symptoms, both of which may be important for some individuals in the recovery process. But this is not universal, and limiting definitions of recovery to the elimination of alcohol consumption and AUD symptoms fails to capture the multidimensional and heterogeneous pathways to recovery that are evident in general population samples, as well as among patients who receive alcohol treatment.^23^

Such a shift in emphasis involves reducing the focus on a pathology-based conception of AUD recovery in favor of incorporating a broader strengths-based, resilience-building approach to behavior change.^44^ Focusing on strengths and building resilience may shift emphasis toward helping people live the life of greatest value to them, which differs from most clinical treatment models and practices that focus on amelioration of disease. Examples of tactics to facilitate this goal include building and strengthening social and community ties, increasing physical activity, and increasing non–substance reinforcement and activities that do not require alcohol use. Clinically, many practitioners using evidence-based treatment approaches are likely already working in alignment with this conceptualization of recovery, which takes a whole person approach to clinical care and focuses on individual strengths, strengthening resilience, and engagement with community support systems. Achieving and maintaining financial stability, as well as housing and food security, is also critically important. Future work is needed to ascertain whether reduced alcohol consumption and remission from AUD symptoms are essential elements in defining recovery or whether a strengths-based model that focuses on well-being and functioning is sufficient to characterize recovery from AUD, or if some combination of relative emphasis on these two broad domains is optimal.

In the AUD field, this shift in emphasis toward improved functioning is exemplied by the concept of “recovery capital” introduced by Granfield and Smith in the context of understanding and promoting natural recovery without treatment.^45^ Their approach focused on building and using internal and external resources (e.g., social, physical, cultural, community) needed for initiation and maintenance of recovery and recognized that recovery capital varies across individuals and is changeable over time. Yet, most American treatment programs remain focused on initiation and maintenance of abstinence, and relatively few address improving well-being, functioning, and life satisfaction. Mutual help groups offer fellowship and support, an important element of recovery capital and positive psychology approaches to behavior change. So the field has made some progress in shifting away from a pathology-based model toward a strengths-based model of AUD recovery. However, these developments have largely been limited to behavioral treatments and recovery attempts outside the context of formal treatment, and many clinical treatment programs have not expanded their focus beyond reducing or eliminating alcohol use and associated symptoms.

Importantly, a shift in focus toward health and well-being should not go too far, as is the case in definitions of recovery that focus heavily on good citizenship and giving back to communities. As discussed by Lancaster, definitions of recovery should never require superhuman changes, and expecting a great abundance of citizenship and other aspirational goals among those in recovery “fail[s] to take into account the differences in the normative and social contexts of people’s lives.”^46^^(p758)^ Some individuals who are in the process of recovery live in societal and cultural systems of disadvantage with ongoing experiences of discrimination that cannot be remediated through individual effort and are made more acute by the stigma of addiction.^47^

More generally, given that alcohol use is legal among adults and consuming alcohol without problems is socially normative behavior, the stigma of AUD is exacerbated when total abstinence from alcohol is a defining feature of health and well-being for one subgroup of individuals (those meeting AUD criteria) and is absent as a defining feature of health and well-being for another much larger subgroup (those not meeting AUD criteria). Moreover, defining recovery by abstinence reinforces the empirically debunked belief that alcohol is harmful only for those with AUD and that they can never drink again. Instead, from a public health perspective, it is crucial to focus on reductions in risks associated with drinking as the primary target for all individuals in the population, not just those with AUD. This is justified given the known deleterious effects of excessive alcohol consumption on health^27,48^ and the well-established prevention paradox, i.e., greater health improvements at the population level will come from even small reductions in alcohol use by risky drinkers with less serious problems, who far outnumber the small minority of individuals who meet criteria for severe AUD.^23^ Furthermore, recent work indicates that presenting information about AUD as existing on a continuum of severity, as compared to a disease model orientation of presence or absence of AUD, was associated with greater problem recognition among non–treatment-seeking heavy drinkers.^49^ Defining AUD and recovery from AUD on a continuum could increase help seeking and/or promote self-change among individuals with AUD.

In conclusion, the authors define recovery as a dynamic process of change characterized by improvements in health and social functioning, as well as increases in well-being and purpose in life. The empirical literature compels this extension of definitions of recovery beyond a singular focus on drinking and symptom reduction to include facilitation and support of improved well-being during active recovery and beyond. Like prior work in the field, this definition is still conceptual, and future work is needed to validate a formal operational definition of recovery that recognizes that positive change often occurs in multiple domains, that recovery may lie along continua, and that there is no singular recovery pathway. The use of standardized instruments that are already widely used in the field—such as the World Health Organization’s Quality of Life measure^50^ and future research on SURE^40^—could move us closer to having a formal operational definition that could be widely useful for individuals with AUD, their families, providers, policymakers, and other stakeholder groups.

## Figures and Tables

**Table 1:** Definitions of Alcohol Recovery

Source	Definition
**Life functioning and context**	
Substance Abuse and Mental Health Services Administration (SAMHSA) (2012)^5^	“a process of change through which individuals improve their health and wellness, live a self-directed life, and strive to reach their full potential” (p. 3)
Recovery Science Research Collaborative (2017)^25^	“an individualized, intentional, dynamic, and relational process involving sustained efforts to improve wellness” (p. 5)
Best et al. (2016)^41^	“a social process, underpinned by transitions in social network composition, that includes the addition of new recovery-oriented groups, where such groups are perceived as attractive, beneficial, and relevant, and involves the concurrent emergence of a new recovery-based social identity” (p. 120)
**Abstinence/Drinking**	
Betty Ford Institute Consensus Panel (2007)^24^	“a voluntarily maintained lifestyle characterized by sobriety, personal health, and citizenship” (p. 222)
Center for Substance Abuse Treatment (2007)^51^	Abstinence; essential recovery (e.g., handling negative feelings without using drugs or alcohol); enriched recovery (e.g., taking responsibility for the things I can change); and spirituality in recovery (p. 1008)
**What do individuals think of recovery?**	
Kaskutas et al. (2014)^6^	Abstinence; essential recovery (e.g., handling negative feelings without using drugs or alcohol); enriched recovery (e.g., taking responsibility for the things I can change); and spirituality in recovery (p. 1008)
Neale et al. (2016)^40^	Substance use, material resources, outlook on life, self-care, and relationships (p. 165)
SAMHSA (2012)^5^	“a process of change through which individuals improve their health and wellness, live a self-directed life, and strive to reach their full potential” (p. 3)
Recovery Science Research Collaborative (2017)^25^	“an individualized, intentional, dynamic, and relational process involving sustained efforts to improve wellness” (p. 5)
Best et al. (2016)^41^	“a social process, underpinned by transitions in social network composition that includes the addition of new recovery-oriented groups, where such groups are perceived as attractive, beneficial and relevant, and involves the concurrent emergence of a new recovery-based social identity” (p. 120)
